# Clinical severity-dependent virulence of *Staphylococcus aureus* from human diabetic foot ulcers drives impaired wound healing in a diabetic murine model

**DOI:** 10.3389/fcimb.2026.1805111

**Published:** 2026-06-05

**Authors:** Keun Seok Seo, Lee M. Nicols, Cara Dale Palmer, K. Taylor Hellmann, D. Ashley Robinson, Alicia K. Olivier, Justin A. Thornton, Khadija Ferdous, Wilson Cooper Brookshire, Joo Youn Park

**Affiliations:** 1 Department of Comparative Biomedical Sciences, College of Veterinary Medicine, Mississippi State University, Mississippi State, MS, United States; 2 Central Surgical Associates, Jackson, MS, United States; 3 Department of Cell and Molecular Biology, University of Mississippi Medical Center, Jackson, MS, United States; 4 Center for Immunology and Microbial Research, University of Mississippi Medical Center, Jackson, MS, United States; 5 Department of Pathobiology and Population Medicine, College of Veterinary Medicine, Mississippi State University, Mississippi State, MS, United States; 6 Department of Biological Sciences, College of Arts and Sciences, Mississippi State University, Mississippi State, MS, United States; 7 Department of Clinical Sciences, College of Veterinary Medicine, Mississippi State University, Mississippi State, MS, United States

**Keywords:** diabetic foot ulcer, diabetic murine model, *Staphylococcus aureus*, virulence, wound healing, macrophage polarization

## Abstract

**Introduction:**

Diabetic foot ulcers (DFUs) are serious complications of type 2 diabetes and are frequently infected with *Staphylococcus aureus*. However, the virulence mechanisms that enable *S. aureus* to adapt to diabetic conditions and drive disease progression remain unclear.

**Methods:**

To investigate the relationship between *S. aureus* virulence and DFU severity, we freshly isolated *S. aureus* from DFU patients with clinical severities ranging from Grade 2 (mild) and Grade 4 (severe). We evaluated their virulence characteristics using in vitro assays and an *in vivo* diabetic pressure wound infection model in diabetic TALLYHO/JngJ mice.

**Results:**

*S. aureus* isolates from Grade 4 DFUs exhibited significantly greater biofilm formation, hemolytic activity, and resistance to oxidative stress compared to Grade 2 isolates. In the in vivo diabetic pressure wound infection model, Grade 4 isolates induced significantly greater tissue necrosis and delayed wound healing, accompanied by sustained expression of pro-inflammatory M1 macrophage-associated genes (*NOS2, IL1B*, and *IFNG*). In contrast, Grade 2 isolates caused minimal tissue necrosis and promoted rapid wound healing with expression of anti-inflammatory M2 macrophage-associated genes (*ARG1, IL4*, and *IL10*).

**Discussion:**

Collectively, these findings demonstrate that highly virulent *S. aureus* strains associated with severe DFUs exhibit enhanced virulence traits that promote host colonization and immune responses skewing macrophage polarization toward a sustained pro-inflammatory M1 phenotype, thereby exacerbating tissue damage and impairing wound healing. These virulence characteristics may serve as functional biomarkers to distinguish pathogenic *S. aureus* from less pathogenic or opportunistic *S. aureus* strains in DFUs.

## Introduction

Type 2 diabetes mellitus (T2DM) is a global health care crisis affecting an estimated 529 million people and is the direct cause of death in about 1.5 million people each year (Perez-Favila et al., 2019; McDermott et al., 2023). Among the complications of diabetes, diabetic foot ulcers (DFUs) are the most common and severe complications which are often further exacerbated by bacterial infections (Iglay et al., 2016; McDermott et al., 2023). *Staphylococcus aureus* is the most frequent pathogen leading to the risk of lower-extremity amputations, with a poor 5-year survival rate of 20-50% (Sohn et al., 2010; Yammine et al., 2020; Raja et al., 2023). The adaptation of *S. aureus* to unique host environments can be facilitated by horizontal gene transfer events and more subtle mutations that occur *in vivo* (McCarthy et al., 2014; Moura de Sousa et al., 2023).

To elucidate the mechanism by which *S. aureus* contributes to severe DFU pathology, several cohort studies have investigated the genetic diversity and virulence characteristics of *S. aureus* isolated from diabetic patients (Dunyach-Remy et al., 2016; Mottola et al., 2016b; Víquez-Molina et al., 2018; Shettigar and Murali, 2020; Lavigne et al., 2021; Wu et al., 2023). However, the results of these studies have been largely inconclusive or inconsistent, likely due to several contributing factors. First, many cohort studies used *S. aureus* isolates from DFUs without access to host metadata such as DFU severity, levels of hyperglycemia, and other immunocompromising conditions such as HIV, cancer, and autoimmune diseases (Lavery et al., 2024; Chaves et al., 2025; Chen et al., 2025). Second, some studies used *S. aureus* isolates that had been archived for extended periods in the freezer or passaged in the laboratory conditions for several years, potentially altering their virulence phenotypes (Luo et al., 2020; Baker et al., 2024). Finally, a lack of T2DM animal models that recapitulate the virulence of *S. aureus* and host-pathogen interactions in DFUs has hindered mechanistic validation of clinical observations (Salgado-Pabón and Schlievert, 2014; Huon et al., 2020).

To address these limitations, we freshly isolated *S. aureus* from swab samples collected in DFU patients ranging from Grade 2 (mild) to Grade 4 (severe), excluding patients with other immunocompromising conditions. To specifically evaluate *S. aureus* virulence, only monomicrobial infection of *S. aureus* was included in the study. Using isolates maintained under minimal laboratory passage in *in vitro* diabetic-relevant conditions, we assessed genetic and functional virulence characteristics of *S. aureus* isolated from Grade 2 and Grade 4 DFUs through complementary *in vitro* assays and an *in vivo* diabetic pressure wound infection model using diabetic TALLYHO/JngJ (TH) mice. Our results revealed marked strain-specific virulence variations. *S. aureus* isolates from Grade 4 DFUs exhibited enhanced functional virulence phenotypes *in vitro* and induced immune responses skewing macrophage polarization toward sustained pro-inflammatory M1 phenotypes, thereby exacerbating tissue damage and impairing wound healing *in vivo*. In contrast, *S. aureus* isolates from Grade 2 displayed attenuated virulence phenotypes *in vitro* and promoted macrophage polarization toward anti-inflammatory M2 phenotypes, facilitating tissue repair *in vivo*. Collectively, the clinical sample collection strategy and *in vitro* and *in vivo* experimental platforms established in this study will provide a robust framework for identifying highly pathogenic *S. aureus* in DFUs which is needed for developing effective therapeutic interventions.

## Materials and methods

### Ethic statement

The protocol for collecting human swab samples from DFU patients was reviewed and approved by the Institutional Review Board for Human Subjects at Mississippi State University (protocol 24-147). Informed consent from all participants were obtained. All animal experiments were reviewed and approved by the Institutional Animal Care and Use Committee at Mississippi State University (protocol 23-150) and conducted in accordance with National Institutes of Health guidelines, the Animal Welfare Act, and U.S. federal law.

### Sample collection

Clinical wound swab samples were obtained from DFU patients at Central Surgical Associates in Jackson, Mississippi, between June 2024 and January 2025 using Starswab II Collection and Transport Systems (14-905-80, Fisher Scientific). Samples were collected without selection bias related to HbA1c levels, age, gender, or race; however, patients with severe immunocompromising conditions such as cancer or autoimmune diseases were excluded to accurately assess the true virulence of *S. aureus* in DFUs. The clinical diagnosis and severity of DFUs were classified and assessed based on the Infectious Diseases Society of America (IDSA) and International Working Group on the Diabetic Foot (IWGDF) Infection Classification by a board-certified surgeon in wound care medicine (Lipsky et al., 2020). The Grade 2 were mild infection in skin or isolated subcutaneous involvement (no deeper tissue involved) and erythema does not extend >2 cm around the wound, while the Grade 4 were severe infection with tissue necrosis and systemic manifestations including core body temperature > 38.0 °C with white blood cell count >12000 or < 4000 cells/mm^3^.

### Bacterial identification and culture conditions

All clinical wound swab samples were cultured on sheep blood agar plate, colistin nalidixic acid (CNA) agar plate (A50BX, Hardy Diagnostics, USA), and MacConkey agar plate (G35, Hardy Diagnostics, USA) at 37°C for 24 to 48 hours. Colonies on sheep blood agar plate or CNA agar plate presumably being *S. aureus* based on morphology were selected and speciated by MALDI-TOF mass spectrometry (VITEK^®^ MS PRIME, USA) at the diagnostic laboratory at the College of Veterinary Medicine, Mississippi State University. Speciation was further confirmed by the PCR amplification of the *nuc* gene specific to *S. aureus* (Costa et al., 2005). To accurately assess the true virulence of *S. aureus* in DFUs, only *S. aureus* isolates from monomicrobial infection were included in the study. Six *S. aureus* isolates from Grade 2 DFU patients, and four *S. aureus* isolates from Grade 4 DFU patients were selected and used for subsequent experiments. These isolates were cultured at 37 °C for 18–24 hours in brain heart infusion medium supplemented with 0.2% glucose-6-phosphate (10127647001, Sigma-Aldrich, USA) (hereafter referred to as BHI-G6P) under anaerobic conditions using the Mitsubishi Anaerobic Pack system to mimic diabetic metabolic conditions as previously described (Seo et al., 2021; Baker et al., 2024).

### Whole genome sequencing and analysis

Multilocus sequence typing (MLST) and virulence factor profiles of 10 *S. aureus* isolates from Grade 2 (n=6) and Grade 4 (n=4) were determined using whole genome sequencing. Genomic DNA was extracted using a DNeasy kit (69504, Qiagen, USA) according to the manufacturer’s instructions. Extracted DNA was quantified with a Qubit HS Assay Kit (Q33231, Thermofisher Scientific, USA), and 150bp paired-end libraries were prepared with a Nextera XT DNA Library Preparation Kit (FC-131-1024, Illumina, USA). Sequencing was performed at the Molecular and Genomics Core Facility of the University of Mississippi Medical Center, using an Illumina NextSeq 2000 instrument. Samples were sequenced to a minimum of 360 × coverage. The reads were demultiplexed and adapter-trimmed with Illumina software and then filtered for minimum base quality ≥ Q13 and length ≥15bp using Trimmomatic v0.39 software (Bolger et al., 2014). The trimmed paired-end reads were assembled *de novo* using shovill v1.1.0 (https://github.com/tseemann/shovill) with the spades assembler v4.0.0 (Bankevich et al., 2012), and tested with CheckM v1.2.3 (Parks et al., 2015) for >95% completeness and <5% contamination. Strain typing of the genome assemblies were performed using mlst v2.23.0 (https://github.com/tseemann/mlst) software. To detect virulence factors, the approach of Challagundla et al. (2018) was used, which involved mapping of reads to the virulence factors database (Chen et al., 2016) clustered and mapped with SRST2 (Inouye et al., 2014) using the min_coverage 60 option.

### Growth analysis

Overnight seed cultures of clinical *S. aureus* DFU isolates were diluted 1: 1000 in BHI-G6P. The 100µL of diluted cultures was dispensed into each well of a sterile 96 well U-bottom plate (Corning, USA) at 37 °C for 24 hours under hypoxic conditions using AnaeroPack Jar (23-246-385, Mitsubishi, Japan). Bacterial growth was determined by measuring the optical density at 600 nm every 3 hours using a Cytation 5 reader (Agilent Technologies, USA).

### Biofilm assay

Ninety-six well flat-bottom plates were coated with 20% human serum overnight at 4 °C (Cardile et al., 2014). Each *S. aureus* strain was cultured in 200 µL of BHI-G6P per well in triplicate at 37 °C for 72 hours under anaerobic conditions using the Mitsubishi Anaerobic Pack system (Baker et al., 2024). After incubation, each well was washed twice with 250 µL of PBS (Invitrogen) and stained with 200 µL of crystal violet staining solution (BBL, USA) for 15 minutes at room temperature. The wells were then washed three times with 250 µL of PBS, and 200 µL of 30% (w/w) acetic acid was added to dissolve the crystal violet (Grossman et al., 2021). Biofilm formation was quantified at OD_570_ using a Biotek Cytation 5 cell imaging multimode reader (Merritt et al., 2005). *S. aureus* ATCC 6538 and *S. epidermidis* ATCC 12228 were used as positive and negative control, respectively. Data are presented as the percentage of biofilm formation, with the mean OD_570_ of the positive control set to 100%. This experiment was repeated three times for each strain.

### Hemolysis assay

Fresh human blood was collected in EDTA containing vacutainer tube (8881340478, Monoject Covidien) and centrifuged (500 × g, 10 min). RBCs were collected and plasma was removed, washed with PBS and diluted to a final 2% RBC in PBS. The exoprotein samples of each *S. aureus* isolate were prepared by collecting and filtering the supernatants of 24-hour cultures grown under *in vitro* diabetic metabolic conditions (Baker et al., 2024). Prepared RBC and each exoprotein sample (50 µL each) were added to each well in a 96 well V bottom plate (3343, Corning, USA) and incubated at 37 °C with 5% CO_2_ for an hour. Sterile medium was used as a negative control, and LukF and LukS of Panton-Valentine leukocidin (PVL) (1 µg of each, 0530-001, 0536-001, IBT Bioservices, USA) was used as a positive control. The incubated plate was centrifugated at 500 ×g for 3 min. The supernatants were transferred to a 96 well plate and the optical density of lysed RBCs was measured at OD_540_ using a Cytation 5 cell imaging multimode reader (Baker et al., 2024). Data are presented as the percentage of hemolytic activity, with the mean OD_540_ of the positive control set to 100%. This experiment was repeated three times for each strain.

### Resistance to reactive oxygen assay

An overnight culture (1 mL) of each *S. aureus* isolate was washed with 1 mL of PBS and resuspended in 1 mL of PBS. A 100 µL aliquot of the bacterial suspension, containing approximately 1 × 10^8^ CFU was added to each tube containing 1 mL of 0, 0.5, or 1 M H_2_O_2_ (386790, Sigma-Aldrich, USA). The cultures were incubated at 37 °C for 2 hours, and the residual H_2_O_2_ was quenched by catalase (219261, Sigma-Aldrich, USA) (Mashruwala and Boyd, 2017). The surviving bacteria were enumerated using a 10-fold serial dilution followed by agar plating. Data are presented as the percentage of survival rate, with the mean bacterial count at 0 M H_2_O_2_ set to 100%. This experiment was repeated three times for each strain.

### *In vitro* bone-marrow derived macrophage polarization assay

Diabetic TH mice (Jackson Laboratory, USA) were sacrificed via cervical dislocation and legs were dissected. Using aseptic technique, bone marrow was extracted from tibia and femur bones following removal of surrounding muscle. To do so, joints were cut using a scalpel and the exposed bone marrow flushed out the ends of the bones using a 25-gauge needle and a 10 ml syringe filled with PBS. Clumps were gently disaggregated using a needle-less syringe and passed through a 70 μm cell strainer. Bone marrow cells were cultured in Dulbecco’s Modified Eagle Medium (DMEM, D6046, Sigma-Aldrich, USA) supplemented with 10% FBS (F2442, Sigma-Aldrich, USA), 1% L-glutamax (35050061, Gibco, USA), and 1% Penicillin/Streptomycin (P4333, Sigma-Aldrich, USA) supplemented with M-CSF (20 ng/mL, M9170, Sigma-Aldrich, USA). On day 6, BMDMs were enumerated and seeded onto 24 well plates at 1x10^6^ cells per well and allowed to rest overnight (Toda et al., 2021). On day 7, the medium was replaced with antibiotic-free high glucose DMEM containing 25 mM glucose to mimic diabetic metabolic conditions (Ayala et al., 2019). BMDMs were infected with PBS or *S. aureus* isolates from Grade 2 and Grade 4 DFUs at a multiplicity of infection (MOI) of 20 for 1 hour. Following infection, wells were then washed three times with PBS and incubated in DMEM containing gentamicin (100 μg/ml, 15710064, Gibco, USA) for 1 hour to kill extracellular bacteria. Wells were then washed three times with PBS and cultured in DMEM containing 25 mM glucose for 24 hours. Uninfected BMDMs were included as controls. At 24 hours post-infection, culture supernatants were collected to assess BMDM cytotoxicity by measuring lactate dehydrogenase (LDH) release using an LDH assay kit (ab65393, Abcam, USA). To calculate the percentage of lactate dehydrogenase (% LDH) indicating cytotoxicity, the LDH activity from cells infected with *S. aureus* was divided by the LDH activity from cells treated with 1% Triton X-100 (maximum release). In some experiments, BMDMs were lysed in 0.01% Triton X-100, and viable bacteria were quantified by plating on BHI agar plates. Total RNA was extracted from the cells using TRIzol (15596026, Invitrogen, USA) for qRT-PCR analyses.

### Pressure wound model using diabetic TH mice

Eight week-old male TH mice were purchased from the Jackson Laboratory and acclimated for 1 week. Mice were randomly assigned to individually ventilated cages under controlled conditions (temperature: 20 °C-25 °C, humidity: 40%-60%) with a 12-hour light cycle. To monitor hyperglycemia, blood glucose levels were measured from tail vein using an AlphaTrak3 monitoring system (Zoetis, USA) to track hyperglycemia. Once TH mice showed sustained hyperglycemia (blood glucose >300 mg/dL for more than 3 consecutive days), they were anesthetized with isoflurane (03060, Vetone, USA) and shaved from the nape of the neck to the tail on the dorsum, and additional hair was removed with depilatory cream. To induce ischemic pressure wounds, the dorsal skin over the scapulae was pinched up, and two neodymium rare earth magnets (0.375-in. diameter × 0.125 in. thick, 3,466 G; K&J Magnetics, USA) were placed to the pinched skin fold for two cycles of 12 hours on and off. Carprofen (2.5–5.0 mg/kg) was administered subcutaneously at induction to reduce pain (Baker et al., 2024). Overnight culture of *S. aureus* grown in the BHI-G6P broth was adjusted to 1 × 10^9^ CFU/mL in PBS, and 10 µL of bacterial suspension was subcutaneously injected in the pressure wound (*n* = 3 mice/group in two independent experiments). The area of the skin lesions was measured daily with a SilhouetteStar camera (ARANZ Medical, USA) and accompanying Silhouette Central data analysis software (ARANZ Medical). Mice were humanely euthanized via CO_2_ inhalation on day 7 or 14 post-infection for RNA analyses, bacterial count analyses, wound measurement, and histopathological examination, as described previously (Park et al., 2015; Seo et al., 2021; Baker et al., 2024).

### Quantitative real time PCR analyses

Total RNA was extracted from *S. aureus*-stimulated BMDM or infected mouse wound tissues (50 mg) using a RNeasy extraction kit according to the manufacturer’s instructions (74104, Qiagen USA). A total of 100 ng RNA was measured by NanoDrop OneC Microvolume UV-Vis Spectrophotometer (ThermoFisher Scientific, USA) and used to perform qRT-PCR using a Superscript one-step qRT-PCR kit (11732020, Invitrogen, USA) (Baker et al., 2024). qRT-PCR was performed on QuantStudio 3 real time PCR system (ThermoFisher Scientific, USA) using the primers listed in **Table 1**. Relative gene expression to the housekeeping gene (TBP) was analyzed by calculating -ΔCT= -[the threshold cycle (CT) of the target gene minus the CT of the reference housekeeping gene control (*TBP*)] (Turabelidze et al., 2010). Relative gene expression was presented by the mean ± SD of 2^-ΔCT^.

**Table 1:** Primer sequences used in the real-time qPCR.

Gene	Forward (5’-3’)	Reverse (5’-3’)
*NOS2*	GAGACAGGGAAGTCTGAAGCAC	CCAGCAGTAGTTGCTCCTCTTC
*IFNG*	CAGCAACAGCAAGGCGAAAAAGG	TTTCCGCTTCCTGAGGCTGGAT
*TNF*	GGTGCCTATGTCTCAGCCTCTT	GCCATAGAACTGATGAGAGGGAG
*IL1B*	TGGACCTTCCAGGATGAGGACA	GTTCATCTCGGAGCCTGTAGTG
*ARG1*	CATTGGCTTGCGAGACGTAGAC	GCTGAAGGTCTCTTCCATCACC
*IL4*	ATCATCGGCATTTTGAACGAGGTC	ACCTTGGAAGCCCTACAGACGA
*IL10*	CGGGAAGACAATAACTGCACCC	CGGTTAGCAGTATGTTGTCCAGC
*TGFB1*	TGATACGCCTGAGTGGCTGTCT	CACAAGAGCAGTGAGCGCTGAA
*TBP*	CTACCGTGAATCTTGGCTGTAAAC	AATCAACGCAGTTGTCCGTGGC

### Histopathological analyses

Pressure wound lesions from diabetic TH mice infected with Grade 2 and Grade 4 *S. aureus* or uninfected control mice were excised at day 7 and day 14. Mouse skin lesions were fixed in 10% neutral-buffered formalin, routinely processed, embedded in paraffin, and sectioned at 5 µm. Sections were stained with hematoxylin and eosin (H&E) and evaluated by a board-certified veterinary pathologist. Wound healing was evaluated using a semi-quantitative scoring system adapted from van de Vyver et al. (2021), assessing re-epithelialization, granulation tissue thickness, keratinization, and dermal fibrosis in samples collected at days 7 and 14 post-infection. Re-epithelialization was scored as absent (no epithelial coverage; score = 0), partial (<95% coverage with visible epithelial tongues; score = 1), or complete (95–100% epithelial coverage; score = 2). Granulation tissue thickness was scored as: absent (no evidence of granulation tissue; score = 0), thin (<1000 µm; score = 1), or thick (>1000 µm; score = 2). Keratinization was defined as the presence of loosely attached or desquamating keratin layers, or a thick parakeratotic stratum corneum on the superficial epidermal surface and was scored as absent (score = 0) or present (score = 2). Dermal fibrosis was scored absent (score = 0) or present (score =2). Higher scores indicate improved wound healing with increased re-epithelialization and granulation tissue formation, whereas lower scores reflect impaired healing. The maximum total wound healing histological score was 8.

## Statistical analyses

Statistical analyses were performed using GraphPad Prism (version 9.4.1, GraphPad, USA). Comparison of uninfected control and infection with *S. aureus* isolates from Grade 2 and Grade 4 DFU for wound measurements, bacterial counts, and qRT-PCR data were conducted using unpaired Student’s *t*-test or one-way ANOVA, followed by Tukey honestly significant difference *post hoc* test. Histology scores were analyzed using the Mann-Whitney test. Differences with a p value <0.05 were considered statistically significant as indicated in each experiment.

## Results

### Demographic and genetic characteristics of *S. aureus* isolates from Grade 2 and Grade 4 DFUs

A total of 61 swab samples were collected from patients with Grade 2 (n=51) and Grade 4 (n=10) DFUs. Among Grade 2 swab samples, 11.8% (6/51) yielded monomicrobial *S. aureus* infection, whereas 27.5% (14/51) yielded polymicrobial infection including *S. aureus* and 15.7% (8/51) yielded polymicrobial infection excluding *S. aureus*. Among Grade 4 swab samples, 40% (4/10) yielded monomicrobial *S. aureus* infection, whereas 40% (4/10) yielded polymicrobial infection including *S. aureus* and 20% (2/10) yielded polymicrobial infection excluding *S. aureus.* Demographic characteristics of patients infected with monomicrobial *S. aureus* infection are summarized in **Table 2**. Notably, *S. aureus* isolates from Grade 2 DFU patients occurred more often with peripheral vascular diseases whereas isolates from Grade 4 DFU patients occurred more often with cardiovascular diseases.

**Table 2:** Demographic characteristics of patients infected with monomicrobial *S. aureus*.

Strain	DFU grade	Gender	Race	Age	HbA1c	Other complications
SA1	2	F	White	82	7.7	Peripheral vascular disease
SA2	2	F	White	73	7.8	Peripheral vascular disease
SA14	2	M	Black	68	10	Peripheral vascular disease
SA21	2	M	White	67	9.3	Peripheral vascular disease
SA28	2	M	White	82	7.2	Cardiovascular
SA39	2	M	Black	40	7.2	Cardiovascular, Peripheral vascular disease
SA23	4	F	White	42	9.2	Cardiovascular, neuropathy
SA27	4	M	White	52	9.3	Cardiovascular, neuropathy
SA33	4	F	Black	69	9.1	Cardiovascular
SA75	4	M	Black	51	7.7	Cardiovascular

**Figure 1:**
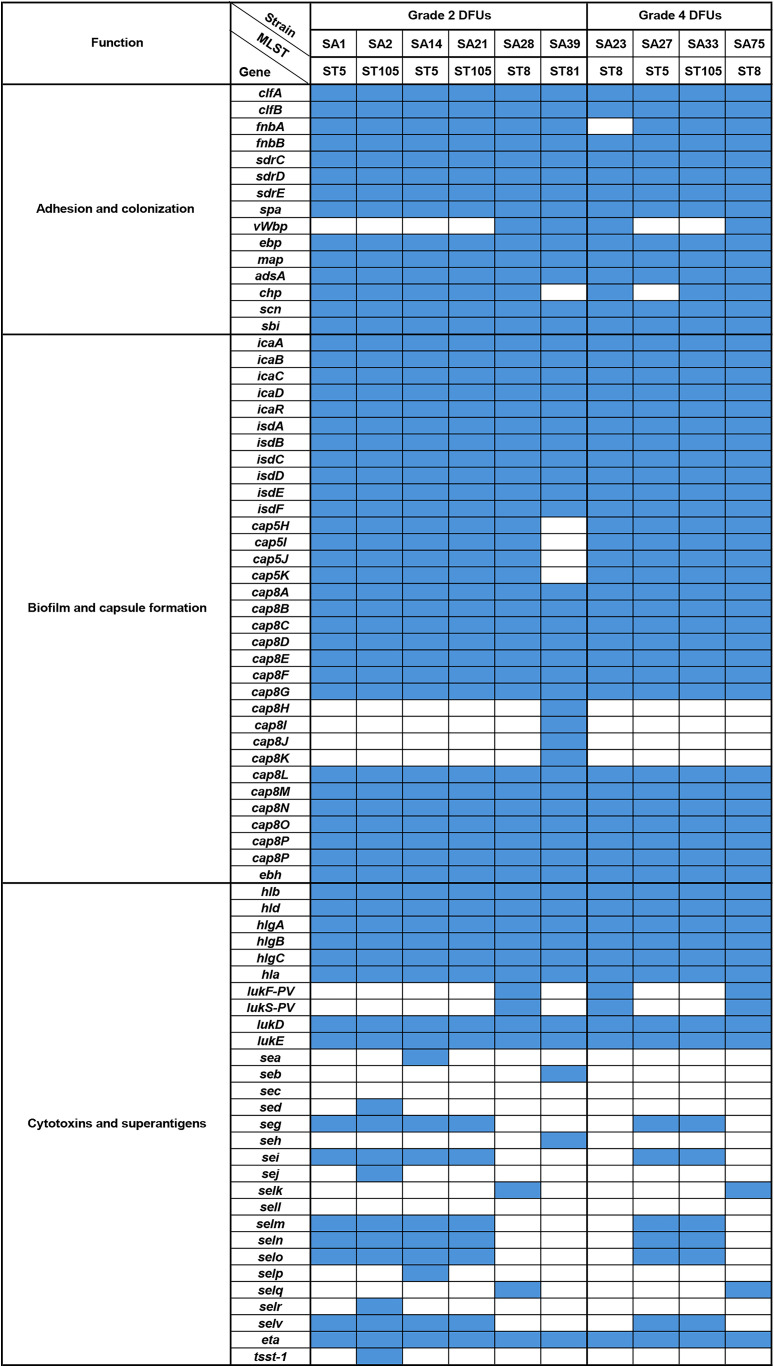
Major virulence factor profiles of *S. aureus* isolates from Grade 2 and Grade 4 DFUs. Blue and white indicate the presence and absence of each virulence factor gene, respectively.

Genetic characteristics of *S. aureus* isolates from Grade 2 (n=6) and Grade 4 (n=4) were determined using whole genome sequencing. The multilocus sequence types (STs) of the Grade 2 isolates were ST5 (n=2), ST105 (n=2), ST8 (n=1), and ST81 (n=1), whereas the Grade 4 isolates were ST5 (n=1), ST105 (n=1), and ST8 (n=2). These isolates represent three major strain lineages within *S. aureu*s including the ST5 and ST105 in clonal complex 5, ST8 in clonal complex 8, and ST81 in clonal complex 1 (Challagundla et al., 2018; Bonn et al., 2023). Major virulence factor profiles are summarized in **Figure 1** and relate to the strain lineage. Overall, no distinct virulence factor profiles were observed between *S. aureus* from Grade 2 and Grade 4 DFUs. Most virulence factors associated with adhesion, colonization, and biofilm formation with exception of von Willebrand factor-binding protein (vWbp) were highly conserved among isolates from both Grade 2 and Grade 4 DFUs. Among cytotoxins, isolates from both Grade 2 and Grade 4 DFUs commonly harbored α-hemolysin, β-hemolysin, γ-hemolysins and leukocidin D and E. Panton-Valentine leukocidin (PVL) were more frequently found in *S. aureus* isolates from Grade 4 (2/4) than Grade 2 (1/6). Greater variability was observed in superantigen profiles. Notably, enterotoxin gene cluster (*egc*) associated superantigens (*seg*, *sei*, *selm*, *seln*, and *selo*) were more frequently identified in isolates from both Grade 2 (4/6) and Grade 4 DFUs (2/4). Exfoliative toxin associated with tissue penetration was highly conserved in both isolates from Grade 2 and Grade 4 DFUs.

### Functional virulence characteristic of *S. aureus* isolates from Grade 2 and Grade 4 DFUs

**Figure 2:**
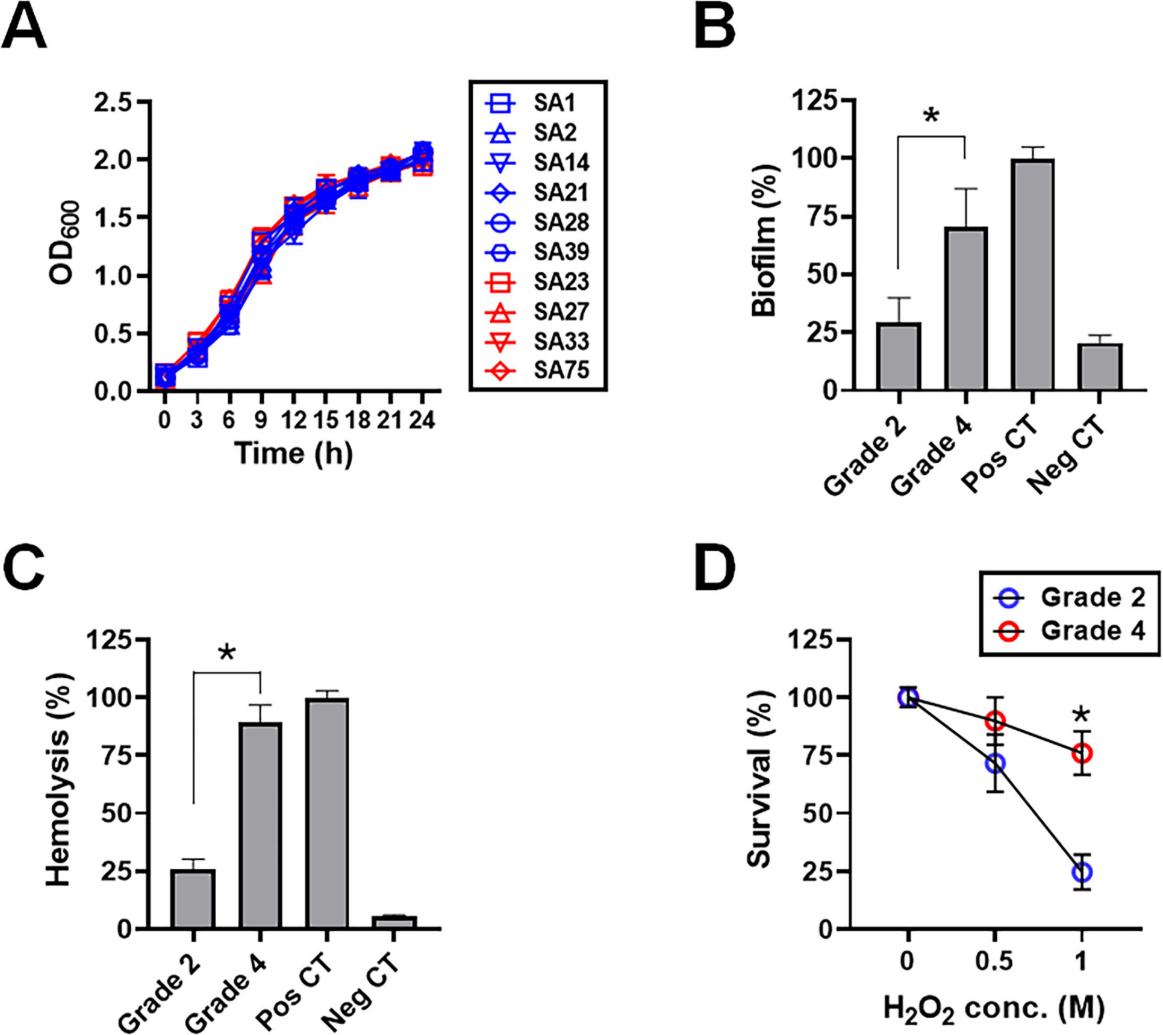
Comparison of growth and virulence phenotypes in *S. aureus* isolates from Grade 2 and Grade 4 DFU patients. **(A)** *S. aureus* isolates from Grade 2 (blue, n=6 strains) and Grade 4 DFUs (red, n=4 strains) were cultured in BHI-G6P broth at 37 °C for 24 hours under anaerobic conditions. Bacterial growth was determined by measuring the optical density at 600 nm (OD_600_). **(B)** *S. aureus* isolates from Grade 2 and Grade 4 DFUs were cultured in BHI-G6P broth at 37 °C for 72 hours under anaerobic conditions and biofilm formation was quantified using the crystal violet staining method measuring absorbance at 570 nm (OD_570_). Data are expressed as a percentage relative to the biofilm positive control (Pos CT) measured with *S. aureus* ATCC 6538 which was set to 100%. The non-biofilm forming strain, *S. epidermidis* ATCC 12228, was used as negative control (Neg CT). **(C)** Human RBCs were incubated with culture supernatants from Grade 2 and Grade 4 *S. aureus* isolates grown in BHI-G6P (2%, w/v) broth at 37°C for 1 hour. A mixture of LukF and LukS (components of Panton–Valentine leukocidin) (1µg of each) were used as positive control (Pos CT). Sterile medium was used as negative control (Neg CT). Following centrifugation, hemolytic activity was accessed by measuring the OD_540_ value of the supernatants and expressed relative to the positive control. **(D)** Overnight culture of Grade 2 and Grade 4 *S. aureus* isolates (100 µL, approx. 1 × 10^8^ CFU) were exposed to 0, 0.5, or 1 M H_2_O_2_ in a final volume of 1 mL and incubated at 37 °C for 2 hours. Viable bacteria were quantified by serial dilution and plating after quenching the residual H_2_O_2_. Survival rates were calculated relative to the 0 M H_2_O_2_ condition, set at 100%. All experiments were performed three independent times with three technical replicates. Data are presented as the mean ± SD. Statistical significance was determined by an unpaired Student’s *t*-test. * p<0.05.

To evaluate functional virulence characteristics of *S. aureus* isolates from Grade 2 and Grade 4, virulence characteristics associated with efficient colonization, penetration, and tissue destruction including biofilm formation, cytotoxicity, resistance to reactive oxygen stress were measured. To minimize functional phenotype alteration during laboratory subculturing, all strains were archived and tested within 2 weeks of initial isolation from blood agar plate (limited to fewer than 3 *in vitro* passages). Although no differences in growth were observed between *S. aureus* isolates from Grade 2 and Grade 4 DFUs (**Figure 2A**), distinct differences were observed in their virulence phenotypes. *S. aureus* isolates from Grade 4 DFUs exhibited significantly greater biofilm formation (70.5 ± 16.3%) than Grade 2 *S. aureus* isolates (29.5 ± 10.8%) (p<0.05) (**Figure 2B**). Similarly, *S. aureus* isolates from Grade 4 demonstrated significantly higher hemolytic activity against human red blood cells (89.3 ± 7.4%) which was 3.4-fold higher than that observed for Grade 2 *S. aureus* (25.6 ± 4.5%) (p<0.05) (**Figure 2C**). In addition, *S. aureus* isolates from Grade 4 showed increased resistance to reactive oxygen stress (H_2_O_2_) in a dose-dependent manner compared with isolates from Grade 2 (**Figure 2D**). At 0.5 M H_2_O_2_, the mean survival rate of *S. aureus* isolate from Grade 4 was 89.8 ± 10.2%, whereas Grade 2 isolates exhibited a survival rate of 71.6 ± 12.3%. At 1 M H_2_O_2_, the mean survival rates of *S. aureus* isolates from Grade 4 DFUs remained high (76.0 ± 9.3%), which was 3.1-fold greater than that of Grade 2 strains (24.6 ± 7.5%) (p<0.05). Taken together, these results indicate that *S. aureus* isolates from Grade 4 possess markedly enhanced functional virulence characteristics associated with efficient colonization, penetration, and tissue destruction, compared with Grade 2 isolates.

### *In vitro* inflammatory response of bone marrow derived macrophages to *S. aureus* isolates from Grade 2 and Grade 4 DFUs

**Figure 3:**
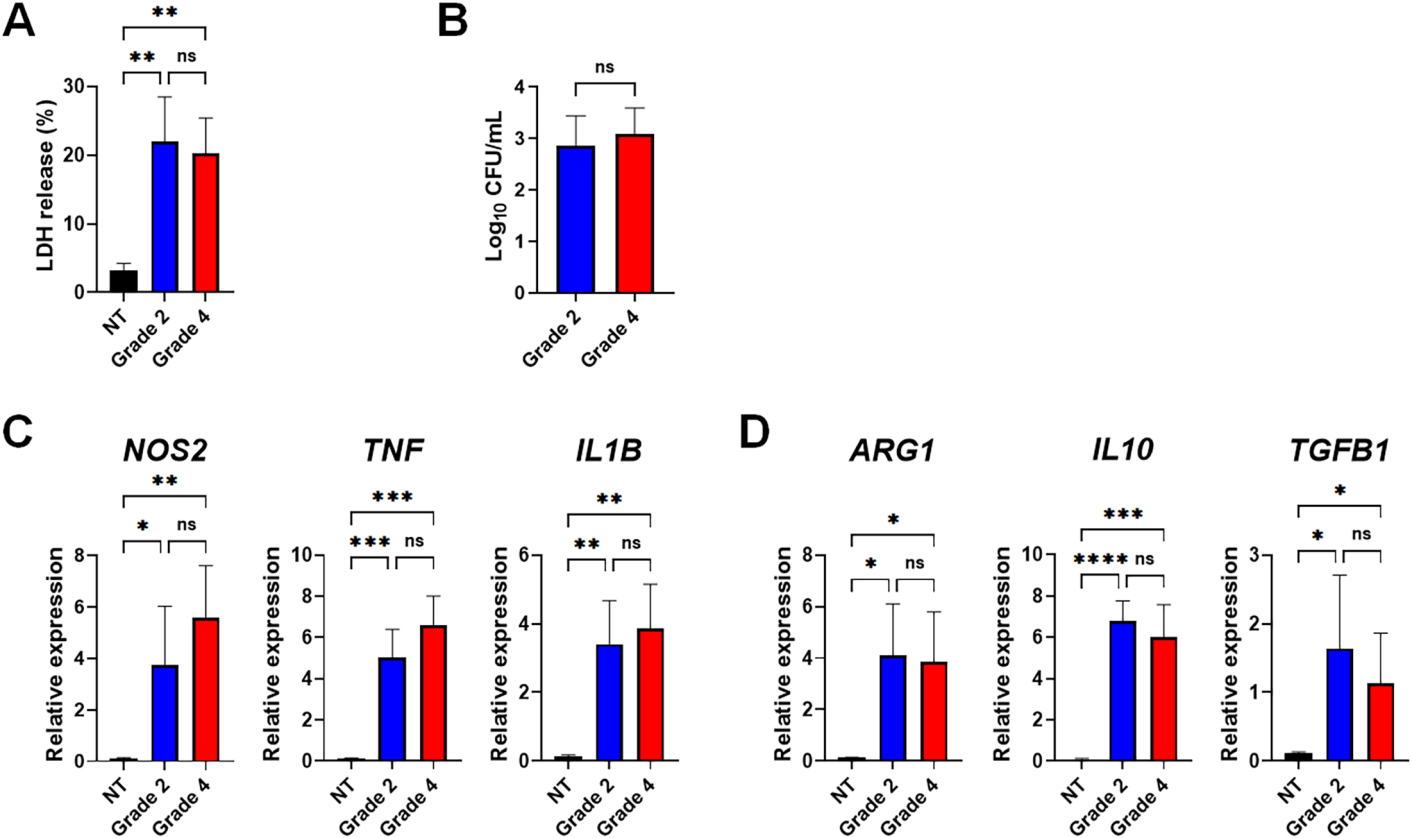
*In vitro* bone-marrow derived macrophage (BMDM) responses to *S. aureus* isolates from Grade 2 and Grade 4 DFU patients. Bone marrow derived macrophages (1×10^6^ cells/ml) from diabetic TH mice were either left uninfected (NT) or stimulated with *S. aureus* isolates from Grade 2 (n=6 strains) and Grade 4 (n=4 stains) DFUs at a MOI of 20 for 24 hours. **(A)** The lactate dehydrogenase release assay of BMDMs infected with *S. aureus* at MOI = 20 for 24 hours. **(B)** Intracellular bacterial survival in BMDMs infected with *S. aureus* isolates at MOI = 20 for 24 hours. Statistical significance was evaluated using an unpaired Student’s t-test. **(C)** Expression of markers associated with M1 (*NOS2*, *TNF*, and *IL1B*) and **(D)** M2 (*ARG1*, *IL10*, and *TGFB1*) macrophage polarization was quantified relative to the *TBP* (-ΔCT) using quantitative real time PCR. All experiments were performed three independent times with three technical replicates. Data are presented as the mean ± SD of 2^-ΔCT^. Statistical significance was determined using ANOVA followed by Tukey's honestly significant difference test. * p<0.05, ** p<0.01, *** p<0.001, **** p<0.0001, n.s, not significant.

Macrophages are central regulators of inflammation, orchestrating their initiation, amplification, and resolution through polarization into pro-inflammatory M1 and pro-resolving M2 phenotypes (Murray et al., 2014; Rath et al., 2014; Rodríguez-Morales and Franklin, 2023; Yan et al., 2024). To characterize inflammatory responses to *S. aureus* isolates from Grade 2 and Grade 4 DFU, bone marrow-derived macrophages (BMDMs) isolated from diabetic TH mice were infected with *S. aureus* isolates from Grade 2 and Grade 4 DFU for 24 hours. Cytotoxicity of BMDMs following infection with *S. aureus* was evaluated by measuring LDH release. Approximately 20% of BMDMs were killed, with no significant difference observed between Grade 2 and Grade 4 isolates (**Figure 3A**). Intracellular survival of *S. aureus* in BMDMs was approximately 0.02%, with no significant difference between Grade 2 and Grade 4 isolates (**Figure 3B**). Transcription of genes associated with M1 (*NOS2*, *TNF*, and *IL1B*) and M2 (*ARG1*, *IL10*, and *TGFB1*) macrophage polarization was then assessed by quantitative real time PCR. Although statistically not significant, BMDMs stimulated with Grade 4 isolates exhibited a trend toward increased expression of M1-associated genes (**Figure 3C**), whereas stimulation with Grade 2 isolates showed a tendency toward elevated expression of M2-associated genes (**Figure 3D**). Uninfected control BMDM (NT) showed minimal expression of genes associated with M1 and M2.

### Enhanced pathogenicity of Grade 4 DFU *S. aureus* isolates drives delayed wound healing independent of bacterial burden

**Figure 4:**
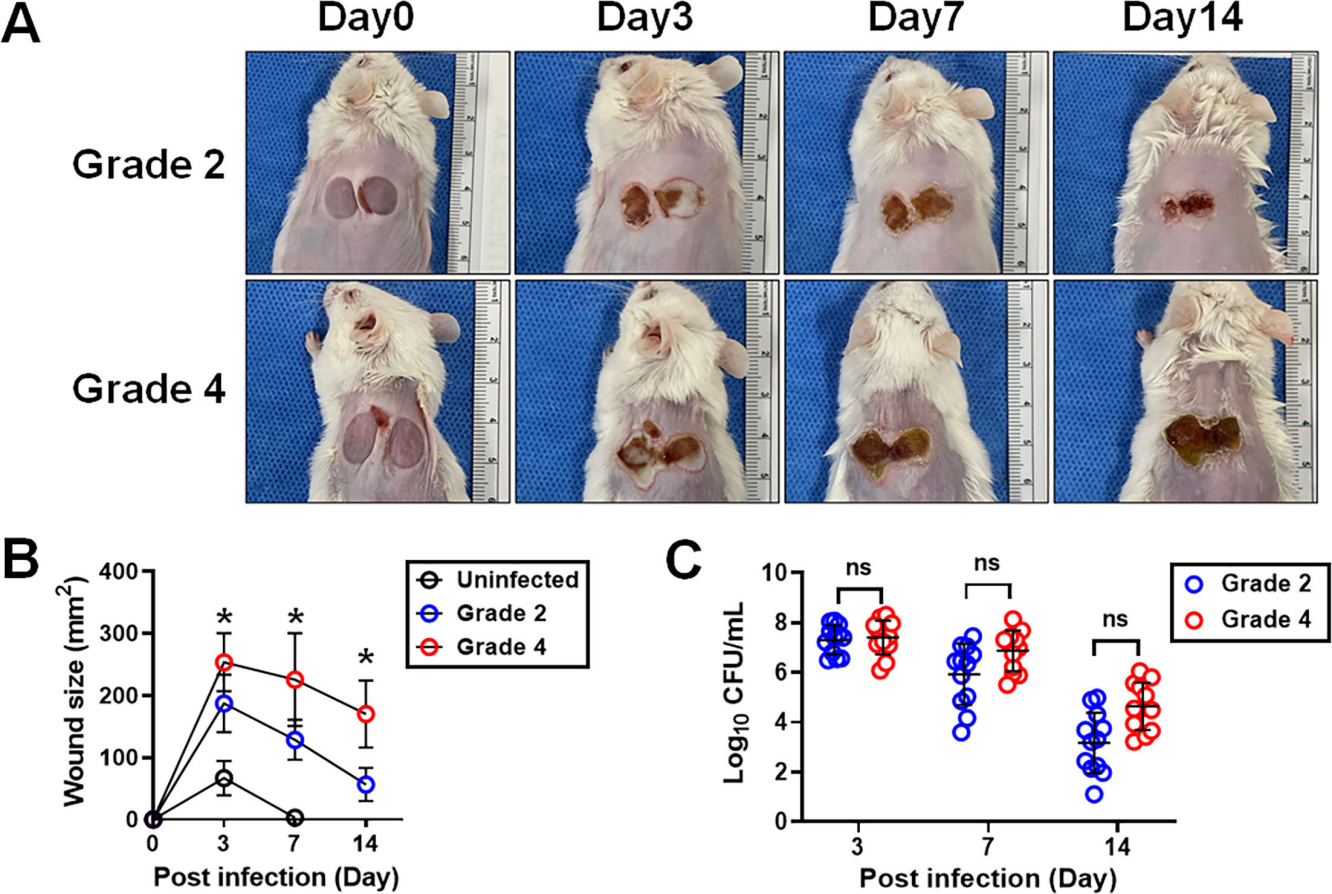
*S. aureus* isolates from Grade 4 DFUs showed severe tissue necrosis in pressure wound in diabetic TH mice than those from Grade 2 DFUs. **(A)** Representative images of pressure wounds infected with *S. aureus* isolates from Grade 2 (n=6 strains) and Grade 4 (n=4 stains) DFUs at 1 × 10^7^ CFU in 10 µL in diabetic TH mice (n=6/strain). **(B)** Quantification of wound area infected with *S. aureus* or uninfected as a control. **(C)** Bacterial burdens at pressure wound measured on day 3, 7 and 14 post-infection. All experiments were performed three independent times with three technical replicates. Data are presented as mean ± SD. Statistical significance was determined by an unpaired Student’s t-test (* p<0.05; ns, not significant).

The pathogenicity of *S. aureus* isolates from Grade 2 and Grade 4 DFUs were assessed using a pressure wound infection model in diabetic TH mice. Briefly, pressure wounds were generated on the dorsal skin using magnetic discs applied in two 12-hour cycles to induce ischemia–reperfusion injury, followed by bacterial inoculation at the wound site. Wound progression was monitored longitudinally, and wound areas were quantified using digital imaging analysis. As shown in **Figure 4A**, wounds infected with *S. aureus* isolates from Grade 4 DFUs exhibited markedly impaired healing compared to those infected with Grade 2 isolates. Quantitative analysis revealed significantly larger wound areas in the Grade 4 group over the course of the experiment (**Figure 4B**), indicating delayed wound closure and increased tissue damage. Uninfected control pressure wounds were completely healed at day 7 (**Figure 4B**).

Despite these differences in disease severity, bacterial burdens at the wound site were not significantly different between the two groups at the examined time points (**Figure 4C**). In both groups, bacterial counts gradually declined over time, suggesting that the observed differences in wound healing were not attributable to differences in bacterial load but rather to isolate-specific virulence characteristics and host response. These findings indicate that *S. aureus* isolates from Grade 4 DFUs exhibited enhanced pathogenic potential that impairs wound healing independently of bacterial burden.

### Differential macrophage polarization gene expression profiles in pressure wounds infected with *S. aureus* isolates from Grade 2 and Grade 4 DFUs

**Figure 5:**
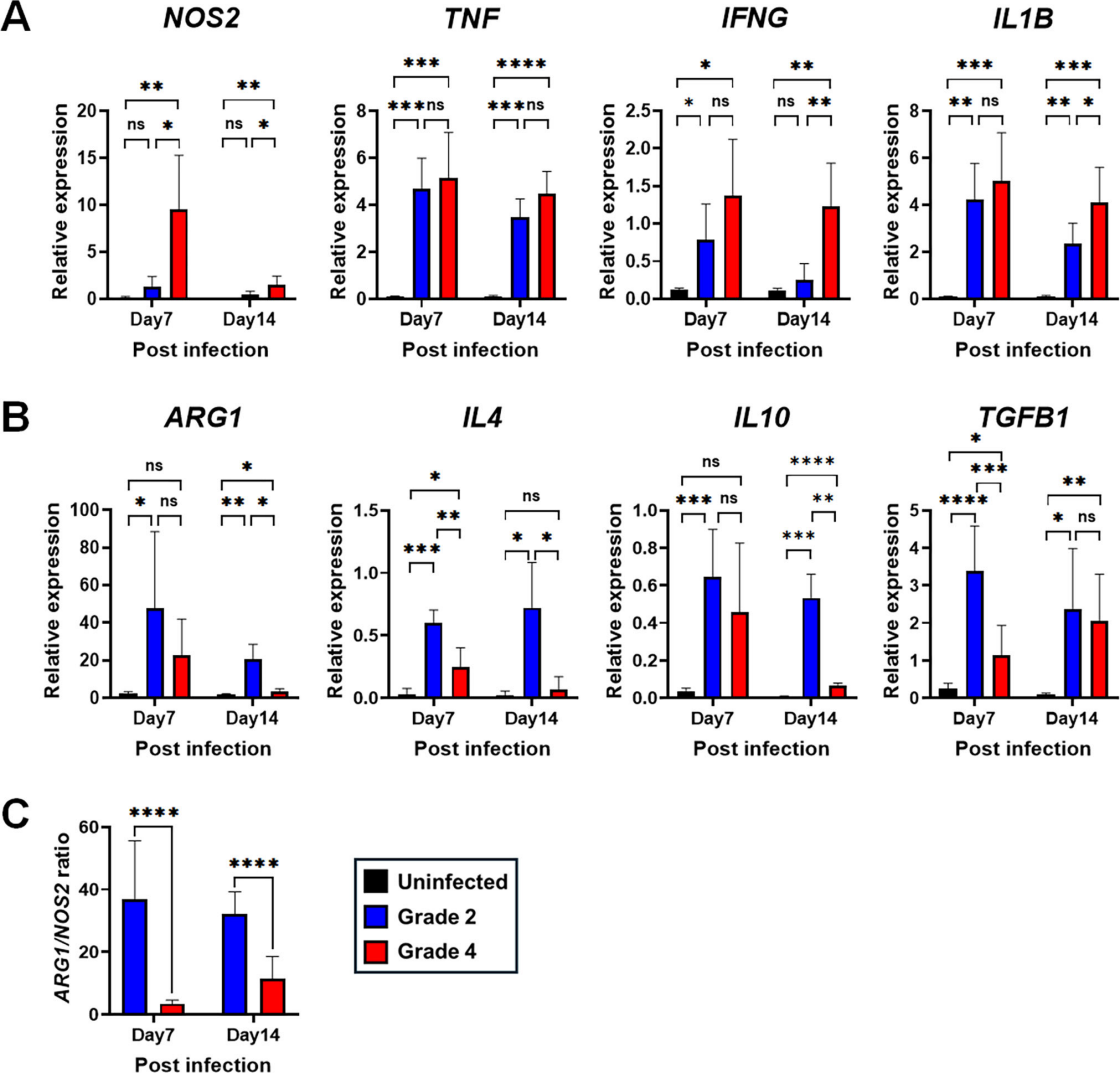
Differential expression of M1- and M2- associated genes in pressure wounds infected with *S. aureus* isolates from Grade 2 and Grade 4 DFUs in diabetic TH mice. **(A)** Expression of markers associated with M1 (*NOS2, TNF*, and *IL1B*) and **(B)** M2 (*ARG1, IL10*, and *TGFB1*) macrophage polarization in pressure wounds infected with *S. aureus* isolates from Grade 2 or Grade 4 DFU or uninfected controls at day 7 and day 14 (n=6/group) was quantified relative to the *TBP* gene (-ΔCT) using quantitative real-time PCR. **(C)** The *ARG1*/*NOS2* ratio in pressure wounds infected with *S. aureus* isolates from Grade 2 or Grade 4 DFU was calculated as an indicator of M1/M2 polarization balance. All experiments were performed three independent times with three technical replicates. Data are presented as the mean ± SD of 2^-ΔCT^. Statistical significance was determined using ANOVA followed by Tukey's honestly significant difference test. * p<0.05, ** p<0.01, *** p<0.001, **** p<0.0001, n.s, not significant.

Since M1/M2 macrophage polarization plays a key role in infection control and wound resolution, expression of markers associated with M1 (*NOS2*, *TNF*, *IFNG*, and *IL1B*) (**Figure 5A**) and M2 (*ARG1*, *IL4*, *IL10*, and *TGFB1*) (**Figure 5B**) macrophage polarization were measured at the pressure wound site infected with *S. aureus* isolates from Grade 2 and Grade 4 DFUs on days 7 and 14 post-infection using qRT-PCR. At day 7, expression levels of the *IL4* (p<0.05) and *TGFB1* (p<0.05) were significantly higher at the pressure wound site infected with *S. aureus* isolates from Grade 2 DFUs, whereas expression of the *NOS2* was significantly higher at wound sites infected with *S. aureus* isolates from Grade 4 DFUs. By day 14, difference in gene expression profiles between *S. aureus* isolates from Grade 2 and Grade 4- infected wounds became more pronounced. At this time point, expression levels of *NOS2*, *IFNG*, and *IL1B*, markers associated with M1 polarization, were significantly higher at the pressure wound sites infected with *S. aureus* from Grade 4 DFUs (p<0.05), whereas expression levels of the *ARG1*, *IL4*, and *IL10*, markers associated with M2 polarization, were significantly higher at the pressure wound sites infected with *S. aureus* from Grade 2 DFUs (p<0.05). This resulted in the *ARG1*/*NOS2* expression ratio of 32.1 ± 7.2 and 11.4 ± 7.1 at wound sites infected with *S. aureus* isolates from Grade 2 and Grade 4 DFUs, respectively, which was significantly different on day 14 (p<0.05) (**Figure 5C**). Although not statistically significant, expression of the *TNF* tended to be higher in *S. aureus* isolates from Grade 4-infected wounds on both day 7 and 14, whereas expression of the *TGFB1* was higher at *S. aureus* isolates from Grade 2-infected wounds at both time points, reaching statistical significance on day 7 (p<0.05) but not on day 14 (p>0.05). Uninfected pressure wound controls showed minimal expression of M1 and M2 associated genes. Collectively, these results indicate that *S. aureus* isolates from Grade 4 DFUs induced prolonged M1 macrophage polarization whereas isolates from Grade 2 DFUs promoted sustained M2 macrophage polarization. Notably, despite no significant difference in bacterial burden between *S. aureus* isolates from Grade 2 and Grade 4-infected wound on day 14 (**Figure 4C**), wound healing was significantly delayed in *S. aureus* isolates from Grade 4-infected wounds (**Figures 4A, B**), suggesting that prolonged M1 macrophage polarization at pressure wound sites infected with *S. aureus* isolates from Grade 4 could contribute to delayed wound resolution.

### Histopathological demonstration of impaired wound resolution in pressure wounds infected with *S. aureus* isolates from Grade 4 DFUs

**Figure 6:**
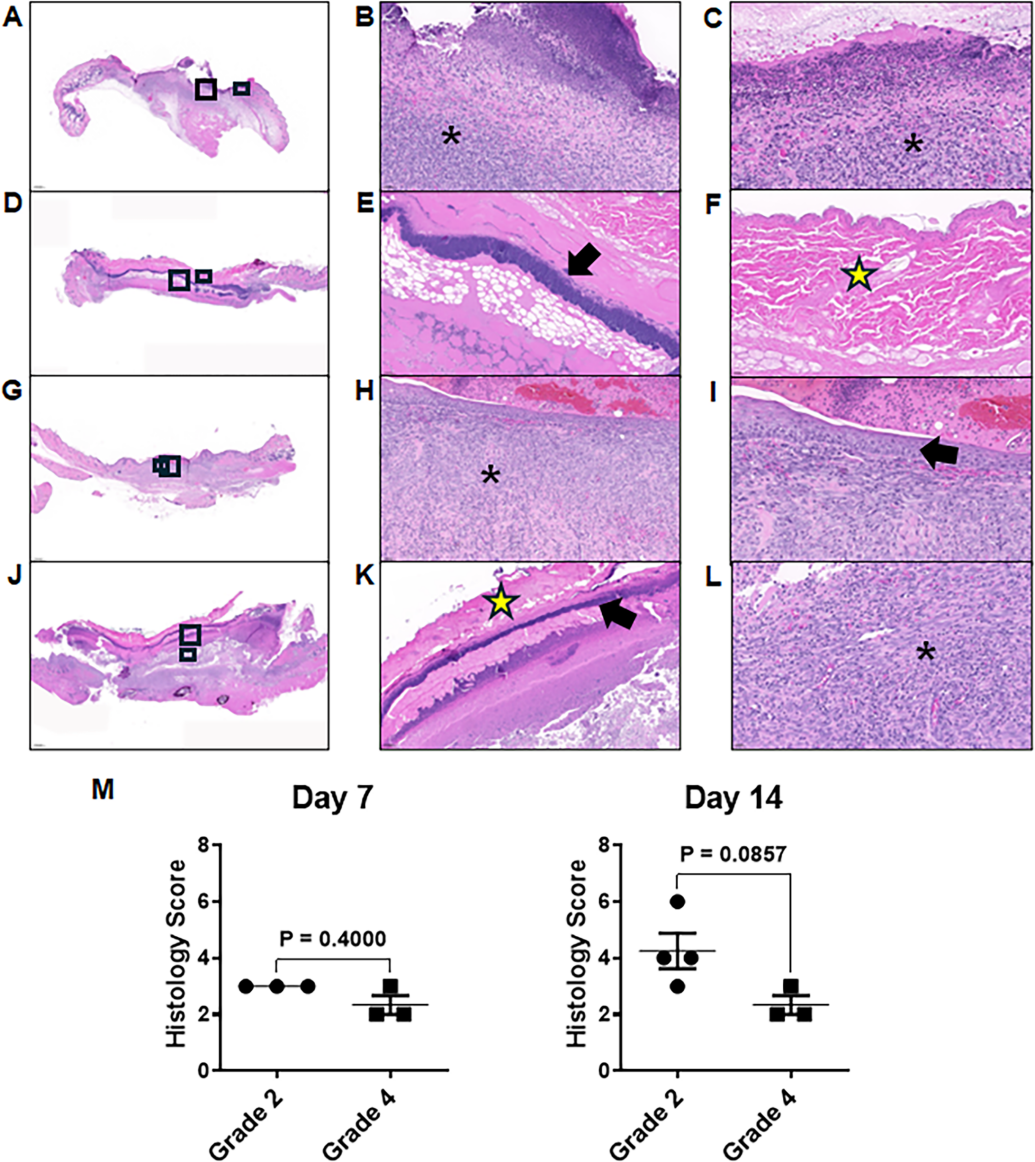
Representative H&E-stained histopathological sections of wound tissues at day 7 and day 14 post-infection with *S. aureus* isolates from Grade 2 and Grade 4 DFUs. **(A–L)** Representative histologic images of infected wounds at days 7 and 14. For each set, the left panel represents the low-magnification view, and the middle and right panels represent higher-magnification images of the boxed regions. **(A-C)** Grade 2-infected wounds at day 7 **(D-F)** Grade 4-infected wounds at day 7 **(G-I)** Grade 2-infected wounds at day 14 show mature granulation tissue (**H**, asterisk) and complete re-epithelialization (**I**, arrow). Boxes in **(G)** indicate regions shown at higher magnification in **(H, I)**. **(J-L)** Grade 4-infected wounds at day 14 show persistent inflammation and delayed wound healing with extensive coagulative necrosis (**K**, star), granulation tissue (**L**, asterisk), minimal re-epithelialization, and abundant bacterial colonies (**K**, arrow). Boxes in **(J)** indicate regions shown at higher magnification in **(K)** and **(L)**. Magnification of H&E sections: **(A, D, G, J)** 2×; **(B, E, H, K)** 10×; **(C, F, I, L)** 20×. **(M)** Semi-quantitative histology scoring at days 7 and 14 post-infection in pressure wounds infected with *S. aureus* isolates from Grade 2 or Grade 4 DFU. Higher scores indicate improved wound healing with increased re-epithelialization and granulation tissue formation, whereas lower scores reflect impaired healing with persistent inflammation and reduced repair. Although Grade 2-infected wounds showed higher scores over time compared to Grade 4-infected wounds, no statistical significance was observed at day 14-posinfection as determined by the Mann-Whitney test (p<0.05). Data were presented as mean ± SD. The maximum total wound healing histological score was 8.

The divergent wound progression between Grade 2 and Grade 4 *S. aureus* infections were analyzed by histopathological evaluation and semi-quantitative histology scoring system (van de Vyver et al., 2021). At day 7, Grade 2-infected wounds showed organized granulation tissue at the wound margins and mixed inflammatory infiltrates, including neutrophils, lymphocytes and sparse macrophages (**Figures 6A–C**), consistent with early repair. At day 7, Grade 4-infected wounds showed extensive dermal coagulative necrosis, dense neutrophilic infiltration, and fibrovascular proliferation surrounding necrotic areas, with minimal re-epithelialization reflected by low histology scores (**Figures 6D–F**). However, despite these histopathological differences, no statistically significant difference in histology scores was observed between Grade 2-infected wounds (histology score: 3.00 ± 0.00) and Grade 4-infected wounds (histology score: 2.33 ± 0.58) at day 7 (**Figure 6M**, p = 0.400). At day 14, Grade 2-infected wounds showed mature granulation tissue, collagen deposition, and epithelial hyperplasia, indicating active tissue remodeling and wound resolution (**Figures 6G–I**). In contrast, Grade 4-infected wounds at day 14 showed persistent neutrophilic inflammation, limited re-epithelization, and delayed granulation tissue maturation which indicate impaired wound resolution compared to Grade 2-infected wounds (**Figures 6J–L**). Although a clear trend toward improved healing in Grade 2-infected wounds compared to Grade 4-infected wounds, the difference in histology scores between the Grade 4-infected wounds (histology score: 2.33 ± 0.58) and Grade 2-infected wounds (histology score: 4.25 ± 1.26) at day 14 did not reach statistical significance (**Figure 6M**, p = 0.0857). Uninfected pressure wounds at day 7 and day 14 (**Supplementary Figure 1**) showed extensive complete epidermal hyperplasia, dermal fibrosis, and loss of adnexal structures, consistent with active repair and structural remodeling of the wound without bacterial-driven inflammation. These wounds showed a histology score of 7, indicating advanced progression of wound healing.

## Discussion

*Staphylococcus aureus* is one of the most prevalent and clinically significant pathogens in DFUs, where it contributes to persistent infection, excessive inflammation, and impaired wound healing. Its ability to form biofilms, evade host immunity, and adapt to the diabetic wound microenvironment makes it a key driver of disease progression and a major barrier to effective treatment (Mottola et al., 2016a; Seo et al., 2021; Nie et al., 2025). To investigate the relationship between clinical severity of DFUs and the virulence of *S. aureus*, we isolated *S. aureus* from 61 swab samples collected from Grade 2 (n=51) and Grade 4 (n=10) DFUs. *S. aureus* was isolated from 28 samples (45.9%, 28/61), including 20 of 51 Grade 2 samples (39.2%) and 8 of 10 Grade 4 samples (80.0%). Among *S. aureus* positive samples, the frequency of monomicrobial *S. aureus* infection was higher in Grade 4 samples (50.0%, 4/8) than in Grade 2 samples (30.0%, 6/20). Consistent with these findings, a previous study reported that *S. aureus* was isolated from 101 of 379 wound samples (26.4%) of which 58 cases (57.4%, 58/101) were monomicrobial *S. aureus* infections and 43 were polymicrobial (Víquez-Molina et al., 2018). Among 58 patients with monomicrobial *S. aureus* infection, infection severity was classified as moderate in 22 cases (37.9%) and severe in 36 cases (62.1%). Collectively, these findings suggest that monomicrobial *S. aureus* infection is more prevalent in severe DFUs.

To determine the relationship between clinical severity of DFUs and the genetic lineages of *S. aureus*, we performed whole genome sequencing analysis. It revealed that major sequence types (ST5, ST8, and ST105) were shared between Grade 2 and Grade 4 DFU isolates. Moreover, no distinct virulence gene profiles were identified that could differentiate Grade 2 from Grade 4 DFU isolates. Despite this genetic similarity, *S. aureus* isolates from Grade 4 DFUs exhibited enhanced virulence phenotypes, including biofilm formation (**Figure 2B**), cytotoxicity (**Figure 2C**), and resistance to reactive oxygen stress (**Figure 2D**). These phenotypes are critical for efficient colonization, tissue penetration, and tissue destruction, suggesting that differences in clinical DFU severity are driven more by phonotypic regulation than by the presence or absence of specific virulence genes that provide more subtle strain-specific adaption to diabetic metabolic conditions.

In the context of adaptation to diabetic metabolic conditions, we demonstrated that elevated extracellular G6P in diabetic tissues activates the HptRS two-component regulatory system, leading to increased cytotoxin production and enhanced intracellular survival, ultimately promoting tissue damage and delayed wound healing in diabetic TH mice (Park et al., 2015; Seo et al., 2021; Baker et al., 2024). Importantly, these virulence phenotypes vary substantially among strains within the same ST, consistent with our observation that both low- and high-severity DFU isolates belong to overlapping strain lineages. Thus, diabetes-associated metabolites likely act as environmental switches that selectively amplify pathogenic potential in certain strains, particularly in Grade 4 DFU isolates, rather than strain lineage alone serving as a predictor of clinical outcome. Collectively, these data support a model in which strain-level regulatory heterogeneity, shaped by diabetes-specific metabolic cues, drives severity of *S. aureus* DFU pathology.

Although the difference in histological score between Grade 2 and Grade 4 at day 14 did not reach statistical significance (p=0.0857), there was a clear trend showing Grade 4-infected wounds exhibited significantly greater tissue necrosis, delayed healing (**Figures 6G–I**), compared to those Grade 4-infected wounds (**Figures 6J–L**). Importantly, differences in wound resolution were not explained by bacterial burden, as both Grade 2 and Grade 4 isolates exhibited similar clearance kinetics (**Figure 4C**). These findings suggest that disease severity is driven not by bacterial load alone but by isolate-specific virulence properties and host-pathogen interactions.

Wound healing is a highly coordinated process consisting of overlapping phases of inflammation, proliferation, and tissue remodeling, in which macrophages play a central regulatory role (Willenborg et al., 2022). During the early inflammatory phase, classically activated (M1) macrophages predominate and produce pro-inflammatory cytokines such as TNF-α, IL-1β, and nitric oxide, which are essential for pathogen clearance and initiation of the healing response (Daley et al., 2010; Willenborg et al., 2012). As healing progresses, macrophages undergo a phenotypic transition toward alternatively activated macrophages, which secrete anti-inflammatory mediators including IL-10 and TGF-β, and promote angiogenesis, extracellular matrix deposition, and tissue repair (Covarrubias et al., 2016; Kimball et al., 2019). Proper temporal regulation of this M1-to-M2 transition is critical for successful wound resolution. In this study, pressure wounds infected with Grade 4 isolates showed gene expression profiles related to proinflammatory M1 macrophage polarization, accompanied by persistent neutrophilic infiltration. In contrast, pressure wounds infected with Grade 2 isolates showed gene expression profiles related to anti-inflammatory M2 macrophage polarization, along with reduced neutrophilic infiltration (**Figures 4**-**6**). These results suggest that delayed wound healing observed in Grade 4 isolates might result from sustained neutrophil recruitment and activation, which can lead to collateral tissue damage through the release of proteases and reactive oxygen species. Concurrently, an impaired transition toward M2-like macrophage phenotypes may limit tissue remodeling and collagen deposition, further contributing to defective wound resolution.

Notably, the differential gene expression related to M1/M2 macrophage polarization was not clearly recapitulated during *in vitro* stimulation of BMDM with *S. aureus* isolates from Grade 2 and Grade 4 DFUs (**Figure 3**). This discrepancy highlights the limitations of *in vitro* systems in capturing the complex of the *in vivo* microenvironment and the cellular interactions required for macrophage functional polarization. Factors such as local cytokine milieu, cell-cell interactions, and extracellular matrix components in conjunction with the chronic metabolic disturbances characteristic of diabetes present *in vivo* such as hypoxia, and diabetic metabolites likely provide critical signals that modulate macrophage behavior during infection and wound healing. This is further supported by our previous findings (Baker et al., 2024) demonstrating that hypoxia induced by pressure wounds significantly delays the resolution of *S. aureus* infection compared to non-hypoxic conditions, likely due to enhanced expression of virulence factors associated with anaerobic fermentation. Collectively, these results underscore the importance of the diabetic pressure wound infection model as a relevant animal model that recapitulates key pathological features of human DFUs caused by *S. aureus*.

This study has several limitations. First, the number of *S. aureus* isolates from Grade 2 and Grade 4 used was relatively small, which may limit the generalizability of the observed associations between clinical severity of DFUs and the virulence characteristics of *S. aureus*. Second, while macrophage polarization was primarily inferred from gene expression and histological features, more comprehensive immunophenotyping and functional analyses, such as flow cytometry and single-cell RNA sequencing across different stages of wound healing, would provide a more precise understanding of the dynamic host-pathogen interactions underlying divergent clinical outcomes. Third, the specific bacterial factors responsible for the observed differences in virulence were not identified and warrant future investigation using integrated genomic, transcriptomic, and functional approaches. Finally, although the murine pressure wound model recapitulates key features of diabetic wound healing, differences between murine and human immune responses should be considered when translating these findings.

We are continuing to isolate and archive clinical *S. aureus* strains from varying degrees of DFUs. As this repository expands, integrated comparisons of transcriptional and translational gene expression profiles between human DFU specimens and corresponding pressure wound infection sites in diabetic mice will enable the identification of conserved pathogenic pathways and host response signatures. This comparative approach will facilitate the discovery of therapeutic targets not only within *S. aureus* itself, but also within host immune pathways dysregulated by *S. aureus* during diabetic wound infection.

## Data Availability

All data generated or analyzed during this study are included in this published article. The sequence reads and assemblies for the 10 isolates have been deposited in the NCBI BioProject database under accession number PRJNA1442169 and are publicly available at https://www.ncbi.nlm.nih.gov/bioproject/?term=PRJNA1442169.

## Supplementary material

Supplementary Figure 1 Histopathological evaluation of uninfected control pressure wounds following magnet treatment. **(A)** Representative histopathological images of uninfected control pressure wounds at day 7 **(A-C)** and day 14 **(D-F)** post-magnet treatment (n=1). Both time points demonstrate extensive epidermal hyperplasia (arrows), dermal fibrosis (*), and loss of adnexal structures, consistent with tissue remodeling and re-epithelialization. Magnification of H&E sections: **(A, D)** 2×; **(B, E)** 10×; **(C, F)** 20×. Histology scores were assessed based on re-epithelialization, granulation tissue thickness, keratinization, and dermal fibrosis in samples collected at days 7 and 14 post-magnet treatment. The histology score of uninfected control pressure wounds at both time points was 7 out of a maximum score of 8.
