# Supplementary figures and images for: Clinical severity-dependent virulence of Staphylococcus aureus from human diabetic foot ulcers drives impaired wound healing in a diabetic murine model
Source: Front Cell Infect Microbiol. 2026 Jun 5;16:1805111. doi: 10.3389/fcimb.2026.1805111 (PMC13279431; doi:10.3389/fcimb.2026.1805111)

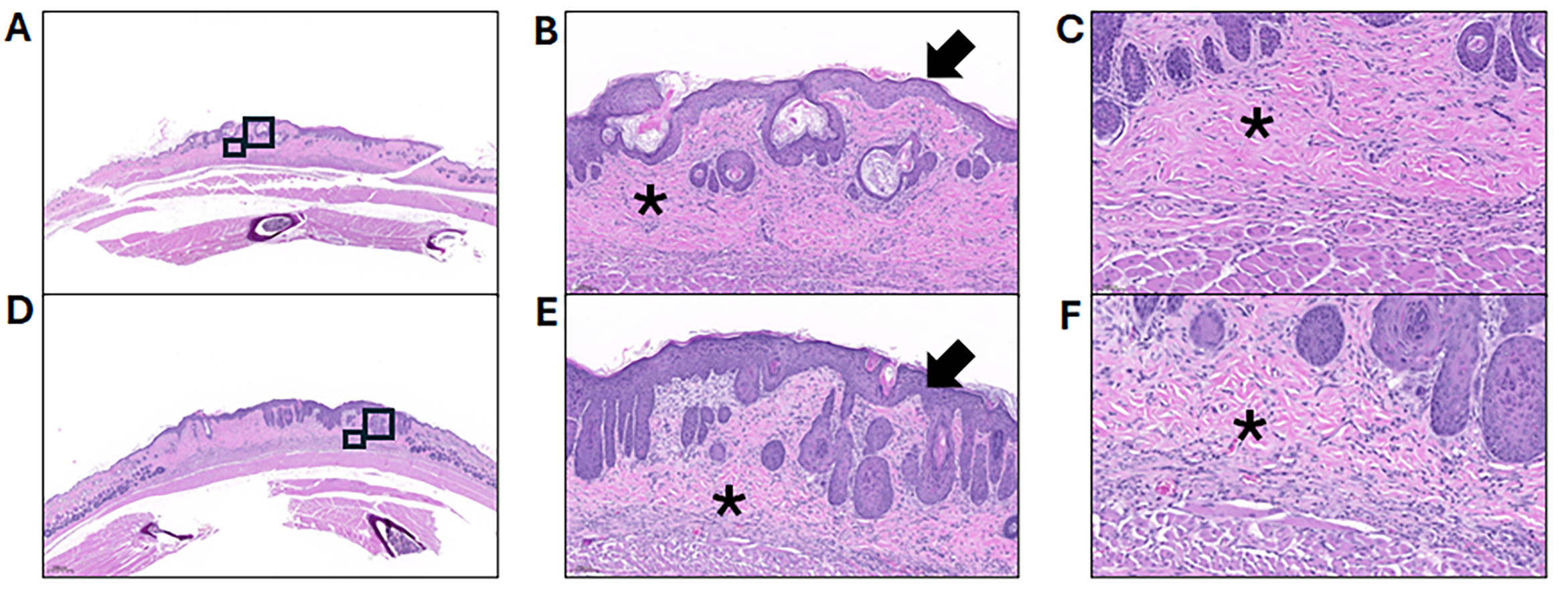

Supplement: Supplementary Figure 1 — Histopathological evaluation of uninfected control pressure wounds following magnet treatment. (A) Representative histopathological images of uninfected control pressure wounds at day 7 (A-C) and day 14 (D-F) post-magnet treatment (n=1). Both time points demonstrate extensive epidermal hyperplasia (arrows), dermal fibrosis (*), and loss of adnexal structures, consistent with tissue remodeling and re-epithelialization. Magnification of H&E sections: (A, D) 2×; (B, E) 10×; (C, F) 20×. Histology scores were assessed based on re-epithelialization, granulation tissue thickness, keratinization, and dermal fibrosis in samples collected at days 7 and 14 post-magnet treatment. The histology score of uninfected control pressure wounds at both time points was 7 out of a maximum score of 8. [file Image1.tif]
